# Assessing knowledge and awareness regarding snakebite and management of snakebite envenoming in healthcare workers and the general population: A systematic review and meta-analysis

**DOI:** 10.1371/journal.pntd.0011048

**Published:** 2023-02-09

**Authors:** Afsana Afroz, Bodrun Naher Siddiquea, Aishwarya Narendra Shetty, Timothy N. W. Jackson, Andrew D. Watt

**Affiliations:** 1 Australian Venom Research Unit, Department of Biochemistry and Pharmacology, Faculty of Medicine, Dentistry and Health Sciences, University of Melbourne, Melbourne, Australia; 2 Department of Epidemiology and Preventive Medicine, School of Public Health and Preventive Medicine, Faculty of Medicine, Nursing and Health Sciences, Monash University, Clayton, Australia; Fundação de Medicina Tropical Doutor Heitor Vieira Dourado, BRAZIL

## Abstract

**Background:**

Snakebite envenoming is a serious and life-threatening medical condition that predominantly affects people living in rural communities across Africa, Asia, and Latin America. As our climate changes, there is a growing concern that negative human–snake interactions will increase. Our ability to prevent and manage snakebite requires effective antivenoms as well as knowledge regarding the prevention and management of snakebite among healthcare workers and affected communities across the globe. This systematic review aims to assess existing levels of knowledge regarding snakebite prevention and management in both healthcare workers and affected communities.

**Methods:**

This review was conducted on studies reporting quantitative measurements to evaluate knowledge and practice regarding snakebite prevention and management published in major databases between 1 January 2000 and 31 December 2021. Random effects modelling was used to obtain the pooled proportion. Heterogeneity (I^2^) was tested, and sensitivity analyses performed.

**Results:**

Out of 3,697 records, 16 studies from 12 countries assessing 7,640 participants were included. Four of the studies were ranked as good quality studies, 9 as fair, and 3 as poor. This study results demonstrated that 56% of the study population answered the knowledge question correctly (95% CI 48% to 63%, *p* < 0.001). High heterogeneity was observed (I^2^ = 97.29%), with marginal publication bias (Egger’s regression test, *p* = 0.0814). Participants had relatively higher knowledge concerning use of antivenom as preferred treatment, followed by snakebite prevention, knowledge of signs and symptoms of snakebite, knowledge of first-aid, and knowledge of treatment. Participants had lower knowledge relating to types of snakes and the identification of snakes.

**Conclusion:**

Adequate knowledge about snakebites and its management among the general population and healthcare workers was 56%. Healthcare workers and communities across Asia showed higher relative knowledge compared to those in Africa and the Middle East. These data suggest that further education is needed in both the general population and among healthcare workers to ensure that appropriate preventative and patient management techniques are being utilised in snakebite endemic regions. Greater local awareness of the risks and appropriate management of snakebite is required to reduce the burden of snakebite mortality and morbidity.

## Introduction

Snakebite is an ecological phenomenon. Snakes bite either to defend themselves from a potential predator or to secure a meal. Human snakebite, therefore, is either defensive, or a case of mistaken identity [1]. Snakebite envenoming is a neglected tropical disease (NTD) that may result in a life-threatening pathology. The burden of snakebite envenoming varies dramatically between regions and disproportionately impacts rural communities in tropical regions of the Global South, particularly Africa, Asia, and Latin America [2–4]. This uneven distribution of the impacts of envenoming are hypothesised to result from occupational practises (e.g., non-mechanised, low-cost farming), poorly constructed houses, and low access to protective clothing (i.e., covered footwear and long pants) that characterise human life in lower socioeconomic rural settings [5–7]. Such practices increase the likelihood of human–snake interaction resulting in snakebite, and the impact on these communities is further compounded by the limited access to healthcare and the untenably high cost of treatment [8], which acts to further exacerbate the poverty cycle.

Recent estimates suggest that between 1.8 to 2.7 million people are envenomed by snakes each year resulting in annual deaths of between 81,000 and 138,000 [6,9,10]. Of those that do survive the initial bite, an estimated 400,000 individuals are left with permanent disfigurement or disability [8]. However, accurate information on the true scale of snakebite in impacted regions has proven difficult to obtain [11]. The lack of mandatory reporting on snakebite envenoming coupled with a historical reluctance of some governments to report accurate data have made it difficult to properly resource and respond to snakebite as a regional health issue. In Nepal, where 90% of the population lives in rural areas, only 480 snake bites and 22 deaths were reported by the Ministry, whereas a community-based study of Eastern Nepal alone reported 4,078 bites and 396 deaths [12]. Similar discrepancies between official and community-reported data have also been observed in India, where a community-based study reported between 40,900 to 50,900 snakebites in 2005, a figure almost 30 times higher than official government numbers [8].

Traditional antivenoms, comprising horse or other animal-derived immunoglobulins, have been the leading treatment for snakebite envenoming for more than a century [6]. These medicines are produced using techniques that have subsequently changed little, besides the refining of methods of inoculation and purification. While the World Health Organization (WHO) provides guidelines for improved standard operating procedures of antivenom manufacture [13], it is also important to assess current levels of awareness in both community groups and healthcare professionals regarding how best to prevent and manage snakebite. Survival following snakebite envenoming is significantly improved by the rapid application of first aid, such as pressure immobilisation bandages, and the administration of life saving antivenoms to neutralise the venom toxins. This dual phase management of snakebite requires both a public aware of local venomous snake species and appropriate snakebite first aid measures and local hospitals with knowledgeable clinicians and readily available and appropriate medicines.

Despite the importance of basic knowledge and awareness concerning snakebite envenoming, multiple studies have found that many healthcare workers in snakebite endemic regions have poor general knowledge of the snakebite crisis [14]. A study by Michael and colleagues reported that doctors in Nigeria had poor knowledge of venomous snakes, snakebite first aid, treatment, and prevention [2]. Similar gaps in baseline knowledge and treatment confidence regarding snakebite patient management were found in doctors across Hong Kong [15], Laos [16], Nepal, and West Bengal [17,18], with the authors of some studies noting that core textbooks regarding snakebite patient management were outdated and provided inaccurate information [18,19].

Among the general population, including at-risk sectors of the population that work outdoors such as farmers, plantation workers, and herdsmen, knowledge of snakebite and appropriate responses are often limited [20,21]. Use of traditional methods such as making incisions, sucking the venom, and application of tight tourniquets [22,23], relying on witchcraft and traditional healers [24], and use of tourniquets [25,26] remain the most commonly used first-aid among general population especially in the rural communities in developing countries. On the other hand, adequate knowledge of snakes, their habits, and the timely and appropriate first-aid can reduce the likelihood and consequences of snakebite among people at high risk of coming into contact with snakes [27].

Socioeconomic and cultural factors influence treatment-seeking behaviours and may lead to individuals bitten by snakes opting for traditional practices rather than hospital care. A lack of money or transportation, or distrust of “Western medicine,” may influence a decision to attend hospital. Compounding this lack of confidence, staff at many health centres are insufficiently trained to treat snakebites, and even if the drug is on hand, it may be too expensive for many victims [28]. Additionally, many antivenoms need to be kept refrigerated to stay stable and effective [6]. In low-resource settings with frequent power cuts, even in cities, keeping them cold can be nearly impossible. Families may seek help instead from a traditional healer, who may apply leaves or ash from burned animal bones, or tie a tourniquet around the bitten limb, which can dangerously restrict blood flow [29]. Some botanical treatments do ease pain and reduce swelling, but they cannot save a victim’s life [29]. The entry into some markets of inappropriate, untested, or even fake antivenom products has further undermined confidence in antivenom therapy generally.

It seems reasonable to conjecture that the burden of snakebite morbidity and mortality may be reduced by a combination of appropriate use of medicines and equipment, adequate training of healthcare workers, and increased awareness of appropriate health-seeking behaviour among the at-risk population. It is thus crucial to gain an understanding of the level of knowledge of the general population and healthcare workers in managing a patient. There is a paucity of data concerning the domain-level knowledge of snakes and snakebite awareness and management in impacted communities and their healthcare workers. This systematic review will draw upon recent studies (selected according to criteria discussed in the Methods section) to assess the level of knowledge regarding prevention and management (outcome) of snakebite among healthcare workers and members of at-risk communities (population).

## Methods

**Fig 1:**
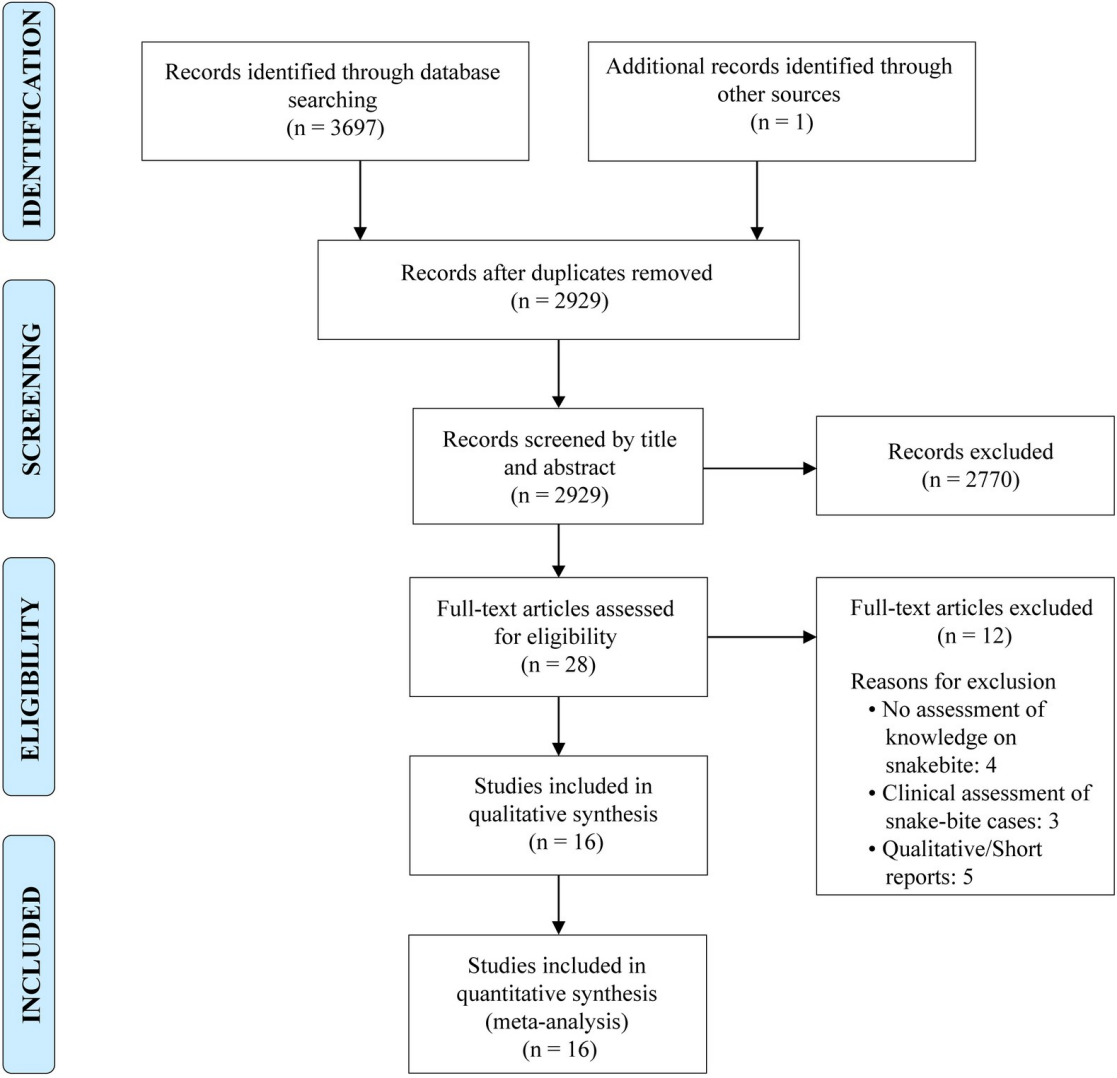
PRISMA 2009 flow diagram.

This systematic review and meta-analysis aimed to assess knowledge and awareness that healthcare workers and the general population have regarding snakebites, and its snakebite prevention and management. The review was conducted according to the Preferred Reporting Items for Systematic Reviews and Meta-analyses (PRISMA) guidelines [30] and was registered on PROSPERO (Reg No: CRD42022377613). The review process is illustrated in **Fig 1** and the PRISMA checklist has been included as supporting information (**S1 PRISMA Checklist**). Ethics approval was not required as this study was based on the published literature.

### Selection criteria

Studies reporting any form of quantitative assessment, measurement, and/or evaluation of knowledge and practice regarding snakebite envenomation and preventive measures, including first-aid, were included. To assess contemporary practises, the selection was limited to articles published in the English language and published in the year 2000 or later. Several studies were identified that reported snake conservation and attitude towards snakes in the absence of specific treatment knowledge and practise, these studies were excluded from the analysis. Qualitative studies, editorials, case reports, and study duplicates were also excluded. To highlight the full state of the field study quality was not a contributing factor to inclusion. Full inclusion and exclusion criteria have been provided in **Table 1**.

**Table 1:** Criteria for study inclusion and exclusion.

Inclusion criteria	Exclusion criteria
1. All published peer-reviewed original studies that reported knowledge and practice of snakebites prevention and management.	1. Studies that did not report knowledge of snakebites.
2. Studies from all geographical areas.	2. Studies that reported snake conservation and attitude towards snakes.
3. Study participants: General people and healthcare workers	3. Qualitative studies, editorials, reviews, case reports, preprints, and study duplicates.
4. Publication year: 1 January 2000 onwards	
5. Language: English	

### Search strategy and study selection

Medline, Embase, CINAHL, and Cochrane databases were searched between 1 January 2000 and 31 December 2021 by 2 authors (BNS and AS) independently, using key terms prepared by a senior librarian at the University of Melbourne. The primary keywords for the search strategy included “knowledge,” “practice,” and “snake-bite.” Searched articles were stored and managed using citation software EndNote X20. A detailed description of the search strategy has been provided as supporting information (**S1 Table**).

Following these searches, BNS and AS independently screened the titles and abstracts of the articles obtained from the search and excluded those articles that did not meet the eligibility criteria. The bibliography of all articles meeting the selection criteria was also screened for additional studies. The final set of 16 articles were selected following a full read and discussion between BNS, AS, and AA. Any disagreement was resolved by the study lead, AA.

### Study outcomes

The primary outcome of this study was to assess knowledge and awareness of snakebite and snakebite management in healthcare workers and the general population that is summarised in the **Table 2**. Although most of the studies uses similar questionnaire to assess the knowledge, in a few of the studies there were differences in questionnaire contents. Thus, the research team grouped the studies into 6 different domains by grouping the common and frequent questions reported by the included studies. This included signs and symptoms of snakebite, snakebite first-aid, treatment, antivenom, and preventive measures associated with the health-seeking behaviour of the general population and the management procedures of the healthcare providers. The secondary outcomes included the determination of estimated knowledge scores by continent, target population, and study quality (i.e., good, fair, poor). Six key knowledge domains were identified to enable more direct comparisons of the primary outcome: knowledge of antivenom as preferred; knowledge of overall treatment; knowledge of signs and symptoms of snakebite; knowledge of first-aid; knowledge of types of snake or identification of snakes; and knowledge of snakebite prevention.

**Table 2:** Description of the outcome measures.

Outcomes	Measures
Overall knowledge	Average knowledge scores reported in each article were extracted and reported as pooled percentages.
Knowledge on types of snakes or identification of snakes	1. The venomous snake’s head is usually oval shaped, with regular teeth marks (false) 2. Correct identification of venomous snakes 3. Correct identification of nonvenomous snakes 4. Identification features of venomous snakes 5. Aware of poisonous and non-poisonous snakes/ability to identify poisonous and non-poisonous snakes
Knowledge on signs and symptoms of snakebite	1. Local bleeding and swelling 2. Severe pain at the site of the bite 3. Nausea and vomiting 4. Drowsiness and weakness 5. Dizziness
Knowledge about first-aid	1. Reassure and calm the patient 2. Immobilise the whole body specially affected part 3. Applying a pressure immobilisation bandage 4. Tourniquet should not be used 5. Bitten site should not be excised 6. Patient should be transported to nearest hospital provided with ASVS
Knowledge about prevention	1. Use a light (torch, flashlight, or lamp) when walking at night 2. Avoid the holes, nests, and other hidden places 3. Do not step on rock or logs/check 4. Wear proper shoes or boots and long trousers 5. Clearing bushes around home
Knowledge on treatment	1. ASV is the only standard/one of/preferred the treatment 2. ASV is specific to snake species 3. Complications of antivenom 4. Treatment of complications 5. Lab tests knowledge
Knowledge about antivenom as a preferred treatment	1. ASV is the only standard/one of/preferred the treatment

### Data extraction and quality assessment

Data from the included articles was independently charted by BNS and AS using Microsoft Excel. Their results were compared and cross-checked by AA and minor discrepancies were resolved through discussion and consensus. The key variables extracted were as follows: publication identifiers (authors, year of publication, journal), study characteristics/methodology (country where the study was conducted, study setting, study design, study population, sample size), participants’ demographics (gender, age, education), and main study findings (prevalence of correct knowledge/mean knowledge score and associated factors). Missing data were sought from study authors, where required.

Study quality was assessed independently by BNS and AS using the quality assessment tool for observational cohort and cross-sectional studies produced by the National Heart, Lung and Blood Institute (NHLBI) [31]. The tool assesses internal validity and risk of bias based on 14 criteria. Each criterion was rated as “yes,” “no,” “cannot determine,” “not applicable,” or “not reported.” The overall quality of the study was then rated as “good,” “fair,” and “poor,” details can be found as supporting information (**S2 Table**). Minor discrepancies were resolved by lead author, AA.

### Data analysis

Quantitative data included the proportion of people with good knowledge toward the outcome across the 6 key domains: knowledge on signs and symptoms of snakebite, knowledge about first-aid, knowledge on overall treatment, knowledge on types of snakes or identification of snakes, knowledge about antivenom as a preferred treatment, and knowledge about prevention. Data were extracted from each study and analysed by the author (AA) and cross-checked by the senior author (AW); any discrepancies were resolved through discussion.

The overall knowledge score in percentages extracted from each study included in this systematic review were reported as pooled percentages using random-effect model. Common and frequent questions reported by the included studies were grouped into 6 knowledge domains including: treatment, signs and symptoms of snakebite, first-aid, snake identification/type, and prevention. The average scores of each domain were then reported as percentages.

The pooled proportion of knowledge and awareness of snakebites and snakebite management was determined using a random-effect model at a 95% confidence interval (CI) [32]. Resulting data were presented in forest plots. Random-effect modelling was used as this method demonstrates better properties in the presence of heterogeneity (if any) by accounting for both within-study and between-study variances [32]. Heterogeneity among studies was tested using the χ^2^-test on Cochran’s Q statistic, which was calculated by means of H and I^2^ indices. The I^2^ index represents the percentage of total heterogeneity across studies based on true between-study differences rather than on chance. I^2^ with a cutoff of ≥75% [33] and a nonsignificant (*p*-value >0.05) result was taken as evidence of no heterogeneity. To identify the possible sources of substantial/considerable heterogeneity, sensitivity analysis was conducted by continent, target population, and study quality (good, fair, poor). Egger’s regression test was used to examine publication bias and the symmetry of the funnel plots was evaluated as previously published [34]. CI was used to evaluate whether differences in prevalence/proportion were statistically significant. As prevalence/proportion cannot fall below 0% or above 100%, the CI is trimmed at 0% and 100% [32]. All statistical analyses were conducted using Stata V.16 (StataCorp., College Station, Texas, United States of America).

## Results

**Fig 2:**
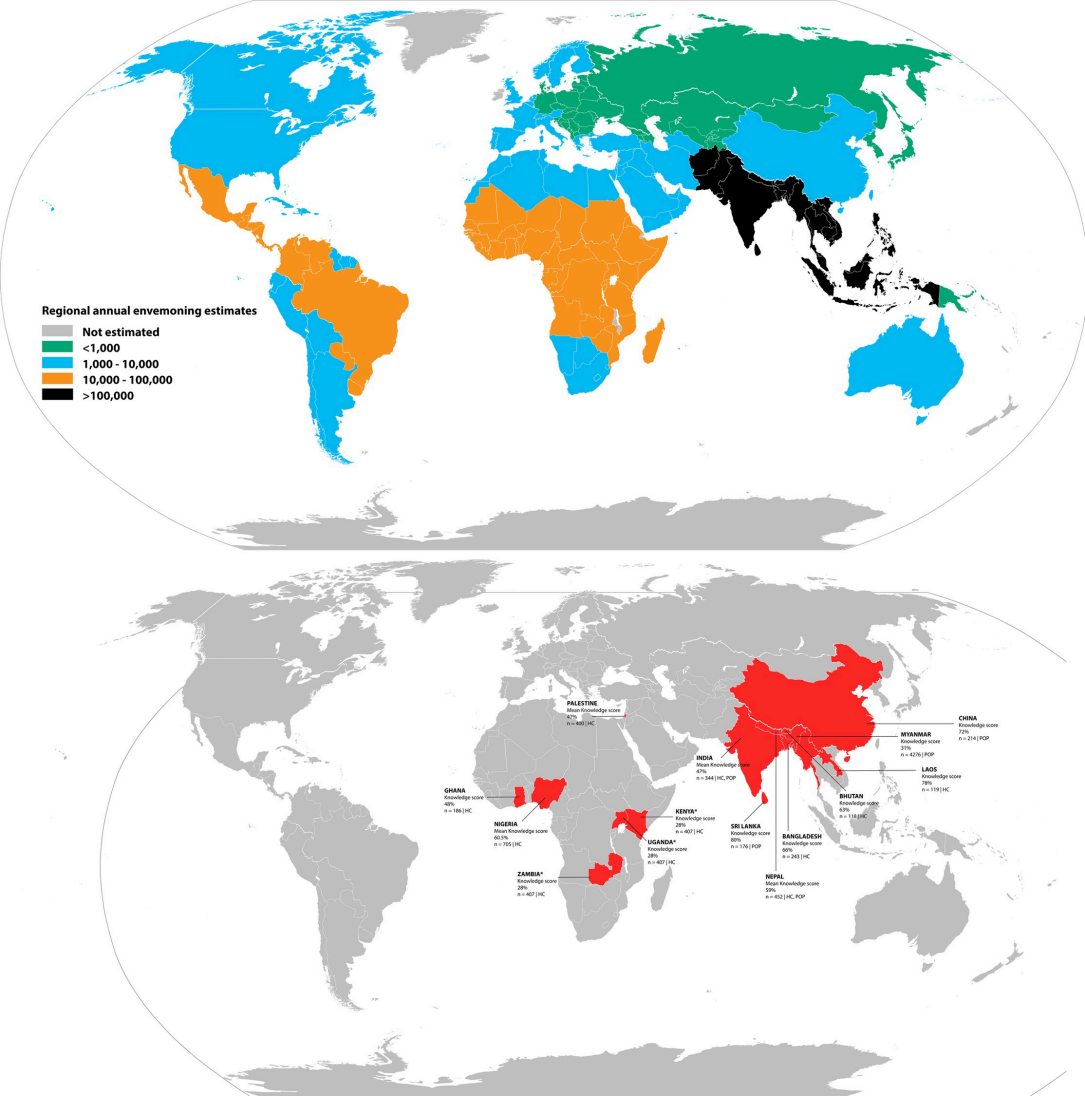
(2A) World map with annual envenoming estimates across the globe vs. (2B) countries with studies included in the current review. Knowledge scores, study populations, and sample size have been provided. For countries with multiple studies, mean knowledge scores have been provided and noted. Study populations include HC = Health Care Workers (clinicians, nurses, medical students, etc.) and POP = General population (the direct link to the base layer of the map: https://commons.wikimedia.org/wiki/File:BlankMap-World.svg).

A total of 3,697 articles, published between 1 January 2000 and 31 December 2021, were retrieved from across the 4 databases and through our additional manual searches. After removing duplicate records and screening by titles and abstracts, 28 articles were included for full-text reading. Of these, 13 articles did not meet the established inclusion criteria and were excluded from further analysis. The countries studied and the regional envenoming estimates are presented in **Fig 2** (2A and 2B). One article was added from manual screening of the bibliography of the included articles.

Sixteen studies [2,4,14,16,18,19,28,35–43] from 12 countries or territories, reporting knowledge of snakebite and snakebite management from 7,640 participants, were included in the quantitative analyses. All studies included in the analysis were online or hospital-based cross-sectional studies.

### Study characteristics

Study features and participant characteristics have been summarised in **Table 3**. Participants were 53.9% male with reported ages ranging from 12 to 90 years. Participants’ education ranged from pre-literate to university-level education. The majority of study participants were from the general population (66.0%; *n* = 5,043), 27.4% were healthcare providers including doctors, nurses, traditional healers (*n* = 2,095), and 6.6% were medical students (*n* = 502). The majority of participants were from Asia (77.8%; *n* = 5,942), 17.0% were from Africa (*n* = 1,298), and 5.2% were from the Middle East (*n* = 400). Of the 16 studies, 4 were ranked as good, 9 as fair, and 3 as poor, in accordance with the NHLBI quality assessment tool.

**Table 3:** Study features and participant characteristics of the studies included in the systematic review.

Study ID	Country	Study settings	Study design	Study period	Study population	Report on	Sample, N	Gender		Age in years Mean (±SD) /range	Overall knowledge score (%)	First author	Publication year
								Male	Female				
1	Bangladesh	Health facilities from 5 districts	Cross sectional		Healthcare providers	Knowledge on sign and symptoms, lab test, complications of antivenom	243				66%	Ahsan and colleagues [40]	2017
2	Ghana	District hospitals in 3 Tongu districts, Volta region	Cross-sectional online	May–June, 2019	Health providers (pharmacists, physician/medical assistants, medical doctors, pharmacy technicians, nurses)	Snake-bite management	186	95	91		48%	Ameadand colleagues [41]	2021
3	Nigeria	Northern Nigeria	Cross-sectional online and offline		Healthcare practitioners (doctors, pharmacists, nurses, and pharmacy technicians)	Anti-snake venom	331	223	108	34.4±8.5	55%	Bala and colleaues [19]	2021
4	India	Dahanu Block in Maharashtra	Cross-sectional	June 2016–October 2018	Healthcare workers (traditional faith healers, snake rescuers, frontline health workers)	Knowledge, awareness, and experience on snakebite and their management	117				56%	Chaaithanya and colleagues [32]	2021
5	China	Field troop in Southeast China	Cross-sectional	July, 2016	General population	Snakebite, prevention, first aid	214	214			72%	Chen and colleagues [42]	2016
6	India	Ten villages in Maharashtra	Cross-sectional	July 2011–June 2012	General population	Type of snake, symptoms, first aid	227			12–90	38%	Chincholikar and colleafgues [43]	2014
7	Laos	Savannakhet Province	Cross sectional	August–September 2015	Healthcare providers	Snakebite management	119	32	87	37+/-10.9	78%	Inthanomchanh and colleages [21]	2017
8	Palestine	An-Najah National University	Cross sectional		Nurse	Snakebite diagnosis and management	200	96	104	20.7+/-1	49%	Kharusha and colleagues [44]	2020
9	Myanmar	144 Villages in Mandalay region	Cross-sectional		General population	Symptoms, prevention, first-aid, health service use and treatment	4276	2142	2134	18–60+	31%	Mahmood and colleagues [45]	2019
10	Nigera	Nine tertiary hospitals in northern Nigeria	Cross sectional		Medical doctors	Knowledge of common venomous snakes, snakebite first aid, snake antivenom treatment and prevention.	374	264	110	35.6±5.8	66%	Micheal and colleagues [2]	2018
11	Kenya, Uganda, Zambia	Twenty-four randomly selected facilities from each of the six survey regions of each country	Cross sectional	March 2018 and November 2019	Healthcare workers	Knowledge of snakebite, and facilities’ snakebite treatment	407			21–30	28%	Ooms and colleagues [46]	2020
12	Nepal		Cross-sectional	January–February, 2013	General population	Snakes, snake bite	150	102	48	15–65+	60%	Pandey and colleagues [24]	2016
13	Bhutan	Ten districts	Cross-sectional, Hospital based	January 2013 to December 2017	Health workers	Knowledge of medical personnel on snakebite management including snake identification	118	73	45	31.9±7.5	63%	Sapkota and colleagues [47]	2020
14	Sri Lanka	Three dry zone districts in Anuradhapura, Jaffna and Vavuniya	Cross-sectional	July–November, 2011	General population (Chena and paddy farmers)	Awareness and perceptions on the venomous snakes in the area, first aid practices for snakebite, snakebite prevention and treatment	176	100	76	42±11	80%	Silva and colleagues [48]	2014
15	Nepal	Gandaki Medical College	Cross sectional	January–May 2018	Medical students	Perception of snake bite	302	153	149		58%	Subedi and colleagues [23]	2018
16	Palestine	An-Najah National University	Cross sectional		Medical students	Knowledge about diagnosis, management and first aid methods of snakebite injuries	200	90	110	22.2 ± 2.4	45%	Sulaiman and colleagues [4]	2020

### Pooled knowledge and awareness of snakebite management across studies

**Fig 3:**
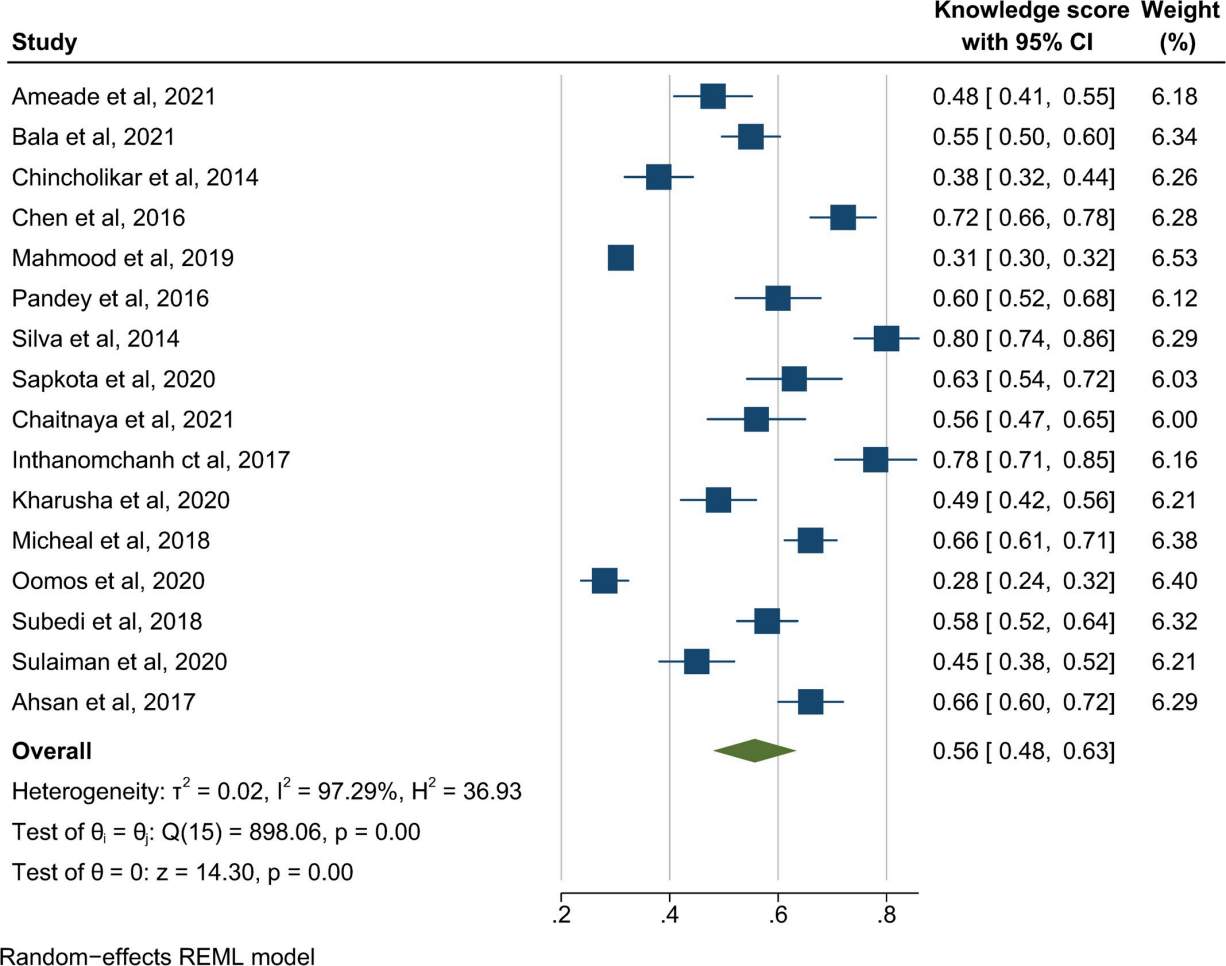
Pooled proportion of knowledge about snakebite, REML-Restricted Maximum Likelihood [2,4,14,16,18,19,28,35–43].

The primary outcome of this study was to assess the knowledge and awareness of snakebite and its prevention and management in healthcare workers and the general population. This included knowledge of signs and symptoms of snakebite, snakebite first-aid, treatment, antivenom, and preventive measures that led to health-seeking behaviour in the general population and the management procedures of healthcare providers. The pooled proportion of people with adequate knowledge of the outcome across the 16 studies is presented in **Fig 3**. These findings demonstrated that 56% of the present study population answered the knowledge question correctly (95% CI 48% to 63%, *p* < 0.001). High heterogeneity was observed (I^2^ = 97.29%), with marginal publication bias (Egger’s regression test, *p* = 0.0814).

### Domain-specific knowledge

To identify possible causes of the substantial heterogeneity observed across the studies, 6 domains of knowledge were identified to enable more direct comparisons: knowledge of overall treatment, knowledge of antivenom as the preferred treatment, knowledge of signs and symptoms of snakebite, knowledge of first-aid, knowledge of types of snakes or identification of snakes, and knowledge of snakebite prevention, study-wise details can be found in **Table 4**.

**Table 4:** Study-wise details of the domains.

	Key domains	Healthcare workers											General people				
		Ameade and colleagues [43]	Bala and colleagues [20]	Sapkota and colleagues [49]	Chaitnaya and colleagues [34]	Inthanomchanh and colleagues [22]	Kharusha and colleagues [46]	Micheal and colleagues [2]	Oomos and colleagues [48]	Subedi and colleagues [24]	Sulaiman and colleagues [4]	Ahsan and colleagues [42]	Chincholikar and colleagues [45]	Chen and colleagues [44]	Mahmood and colleagues [47]	Pandey and colleagues [25]	Silva and colleagues [50]
**1**	**Knowledge on types of snake/identification of snakes**					√*											
a	The venomous snake’s head is usually oval shaped, with regular teeth marks (false)													√			
b	Correct identification of venomous snakes			√				√								√	
c	Correct identification of nonvenomous snakes															√	
d	identification features of venomous snakes			√			√										
e	Aware of poisonous and non-poisonous snakes/ability to identify snakes				√						√		√				√
**2**	**Knowledge on symptoms of snakebite/what are the symptoms of snakebite**												√*				
a	Local bleeding and swelling			√		√	√	√			√	√		√	√		
b	Severe pain at the site of the bite			√			√				√	√		√	√		
c	Nausea and vomiting			√			√				√	√		√	√		
d	Drowsiness and weakness			√		√	√							√	√		
e	Dizziness			√							√	v		√	√		
**3**	**Knowledge on first aid**				√*												
a	Reassure and calm the patient	√		√			√				√			√			
b	Immobilise the whole body specially affected part	√				√		√					√	√	√	√	√
c	Applying a pressure immobilisation bandage	√					√			√	√			√	√	√	
d	Tourniquet should not be used			√		√	√	√		√	√		√	√	√	√	√
e	Patient should be transported to nearest hospital provided with ASVS						√			√	√					√	
f	Bitten site should not be excised					√	√	√		√	√			√	√	√	√
**4**	**Knowledge on prevention of snakebite**																
a	Use a light (torch, flashlight, or lamp) when walking at night							√						√	√		√
b	Avoid the holes, nests, and other hidden places			√				√						√			
c	Do not step on rock or logs/check													√	√		
d	Wear proper shoes or boots and long trousers			√				√						√	√		√
e	Clearing bushes around home			√											√		√
**5**	**Knowledge on treatment**																
a	ASV is specific to snake species	√	√					√									
b	Complications of antivenom	√	√	√		√	√				√	√					
c	Treatment of complications	√	√			√											
d	Lab tests knowledge	√		√			√		√		√	√					
**6**	**Knowledge on antivenom as the preferred treatment**																
a	ASV is the only standard/one of/preferred the treatment for snake envenoming	√	√												√	√	√

* The knowledge was assessed on the overall topic; no specific question was assessed.

**Fig 4:**
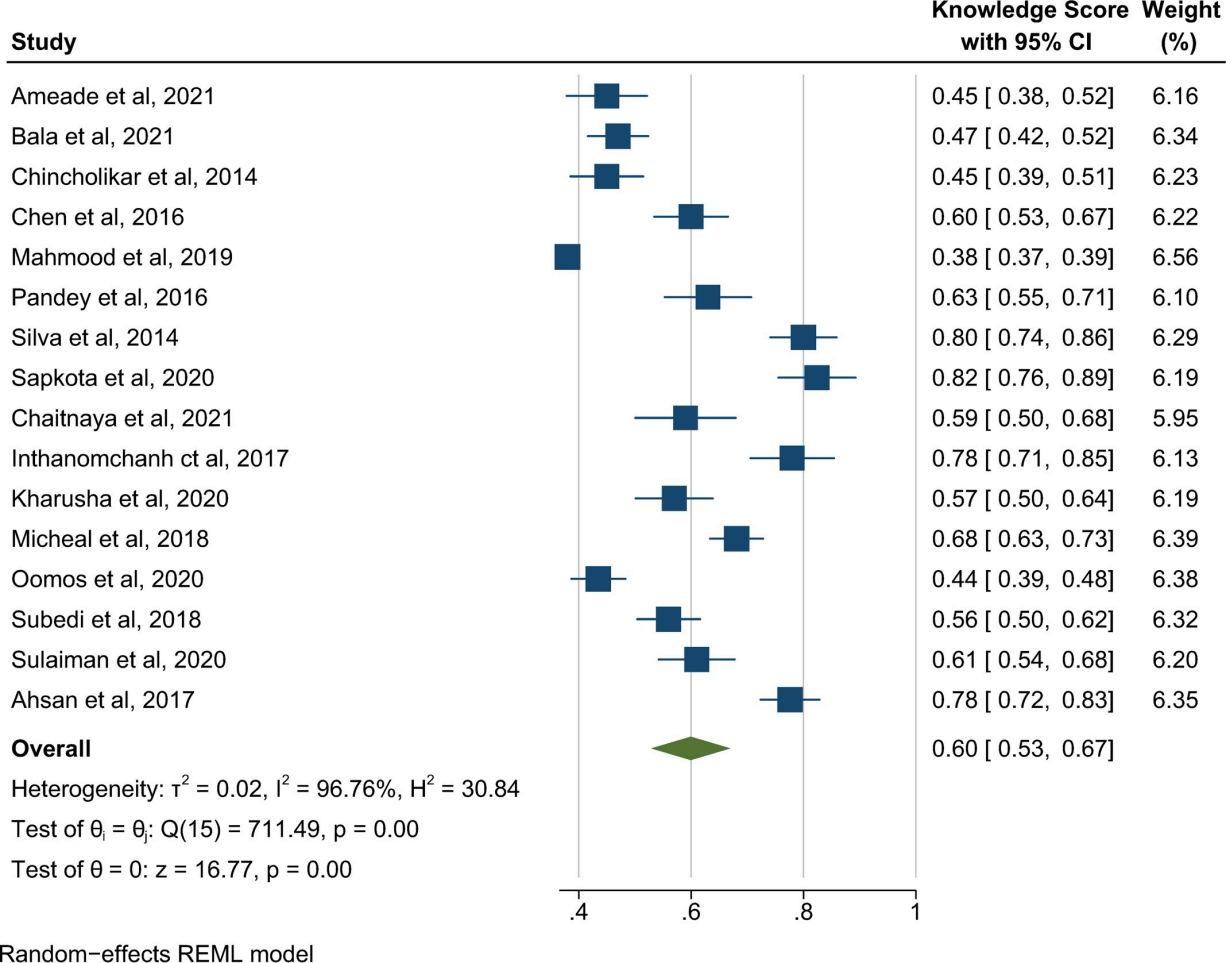
Pooled proportion of knowledge about snakebite of the 6 key domains, REML-Restricted Maximum Likelihood [2,4,14,16,18,19,28,35–43].

Fig 4 represents the pooled prevalence of 60% of good knowledge (95% CI 53% to 67%, *p* < 0.001) in the total of 6 key domains. Heterogeneity was high (I^2^ = 96.76%) and publication bias was observed for the total score of the 6 identified domains (Egger’s regression test, *p* = 0.149) in the studies. A summary of the pooled knowledge scores obtained by the sample for each of the knowledge domains relevant to snakebite management has been provided in **Table 5**.

**Table 5:** Pooled knowledge scores obtained by the sample for each of the knowledge domains.

Key domains	No of studies	Estimates score (%) (95% CI), p-value	I^2^	Egger test (*p*-value)
Knowledge on types of snake/identification of snakes	10	0.54 (0.46, 0.63), <0.001	93.27	0.569
Knowledge on symptoms of snakebite/what are the symptoms of snakebite	9	0.66 (0.52, 0.81), <0.001	99.06	0.129
Knowledge about first aid	13	0.57 (0.46, 0.67), <0.001	98.18	0.738
Knowledge about prevention	5	0.73 (0.52, 0.93), <0.001	99.71	0.235
Knowledge about overall treatment	9	0.56 (0.47, 0.66), <0.001	94.87	0.375
Knowledge about antivenom as a preferred treatment	5	0.78 (0.65, 0.92), <0.001	98.55	0.054
**Overall**	16	0.60 (0.53, 0.67), <0.001	96.76	0.149
**General population**				
Knowledge on types of snake/identification of snakes	4	0.54 (0.33, 0.75)	97.47	0.907
Knowledge on symptoms of snakebite/what are the symptoms of snakebite	3	0.52 (0.12, 0.92)	99.63	0.001
Knowledge about first aid	5	0.46 (0.34, 0.58)	96.33	0.392
Knowledge about prevention	3	0.68 (0.39, 0.98)	99.66	0.797
Knowledge about treatment	-	-	-	-
Knowledge about antivenom as a preferred treatment	3	0.77 (0.55, 0.98)	98.91	0.001
**Healthcare providers**				
Knowledge on types of snake/identification of snakes	5	0.55 (0.48, 0.62)	78.42	0.164
Knowledge on symptoms of snakebite/what are the symptoms of snakebite	4	0.73 (0.64, 0.83)	92.29	0.001
Knowledge about first aid	5	0.65 (0.47, 0.83)	98.06	0.437
Knowledge about prevention	2	0.79 (0.42, 1.00)	99.30	1.000
Knowledge about treatment	9	0.56 (0.47, 0.66)	94.87	0.375
Knowledge about antivenom as a preferred treatment	2	0.81 (0.61, 1.00)	97.91	1.000

**Fig 5:**
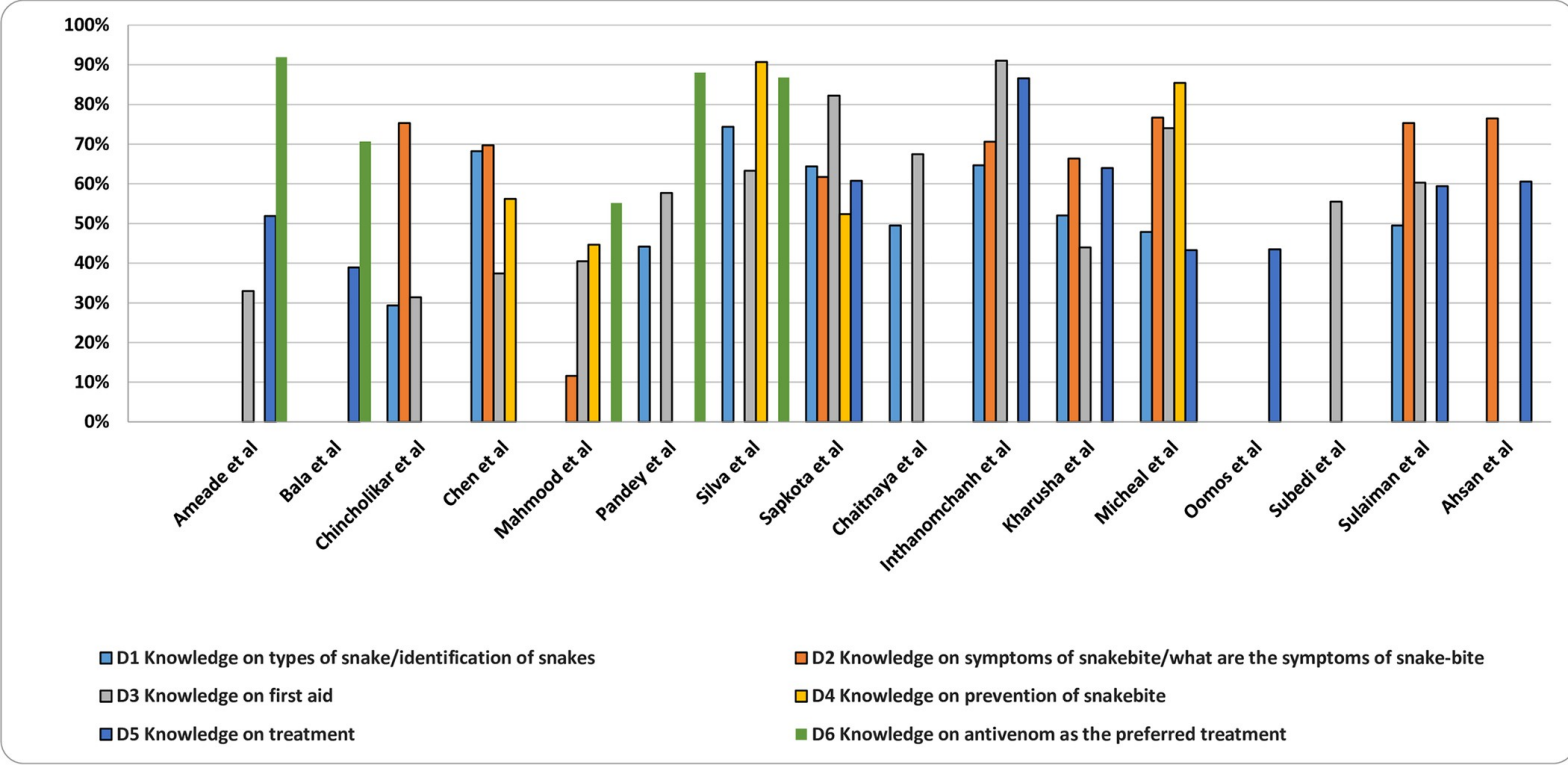
Knowledge scores on snakebite of selected domains [2,4,14,16,18,19,28,35–43].

Participants had relatively higher knowledge concerning use of antivenom as preferred treatment (78%, CI 65% to 92%), <0.001, I^2^ = 98.55), followed by snakebite prevention (73%, CI 52% to 93%, *p* < 0.001, I^2^ = 99.71), knowledge of signs and symptoms of snakebite (66%, CI 52% to 81%, *p* < 0.001, I^2^ = 99.06), knowledge of first-aid (57%, CI 46% to 67%, *p* < 0.001, I^2^ = 98.18), and knowledge of treatment (56%, CI 47% to 66%, *p* < 0.001, I^2^ = 94.87). Participants had lower knowledge relating to types of snakes and the identification of snakes (54%, CI 46% to 63%, *p* < 0.001, I^2^ = 93.27). High heterogeneity and publication bias was observed in all these subgroup analyses. The specific domain wise proportion of good knowledge is detailed in **Fig 5**.

### Sensitivity analysis

Sensitivity analysis was conducted to identify possible sources of substantial/considerable heterogeneity. This analysis was conducted by continent, target population, and study quality (i.e., good, fair, poor), respectively. The results are presented in **Table 6**.

**Table 6:** Sensitivity analysis for the overall pooled knowledge score and domain specific total knowledge score.

Variable	No of studies	Estimates score (%) (95% CI*), *p*-value	I^2^	Egger test (*p*-value)
**Total knowledge score**				
**Continent**				
Africa	4	0.49 (0.33, 0.65), <0.001	97.24	0.819
Asia	10	0.60 (0.50, 0.70), <0.001	97.3	0.105
Middle East	2	0.47 (0.42, 0.52), 0.42	0.03	
**Target population**				
Healthcare workers	9	0.56 (0.47, 0.66) <0.001	95.21	0.314
General people	5	0.56 (0.37, 0.75), <0.001	98.69	0.228
Medical students	2	0.52 (0.39, 0.64), <0.001	87.52	
**Study quality**				
Good	4	0.58 (0.50, 0.65), <0.001	82.39	0.512
Fair	9	0.54 (0.42, 0.66), <0.001	98.11	0.064
Poor	3	0.59 (0.38, 0.79), <0.001	97.05	0.009
**Domain specific total knowledge score**				
**Continent**				
Africa	4	0.51 (0.40, 0.62), <0.001	94.47	0.482
Asia	10	0.64 (0.54, 0.73), <0.001	97.2	0.163
Middle East	2	0.59 (0.54, 0.64), 0.42	0.04	
**Target population**				
Healthcare workers	9	0.62 (0.52, 0.72), <0.001	95.88	0.755
General people	5	0.57 (0.43, 0.72), <0.001	97.7	0.222
Medical students	2	0.58 (0.53, 0.63), <0.001	19.83	
**Study quality**				
Good	4	0.59 (0.50, 0.68), <0.001	89.26	0.981
Fair	9	0.60 (0.49, 0.71), <0.001	97.71	0.101
Poor	3	0.61 (0.42, 0.79), <0.001	96.42	0.091

*CI: Confidence Interval

The proportion of good knowledge varied between continents. Participants from Asia (60%, CI 50% to 70%, I^2^ = 97.30, *p* < 0.001) had better knowledge compared to Africa (49%, CI 33% to 65%, I^2^ = 97.24, *p* < 0.001), and the Middle East (47%, CI 42% to 52%, I^2^ = 0.03%, *p* = 0.42). However, the difference was not significant as the respective CIs overlapped. The proportion of knowledge was similar and slightly higher among the general population (56%, CI 37% to 75%, I^2^ = 98.69, *p* < 0.001) and the healthcare providers (56%, CI 47% to 66%, I^2^ = 95.21, *p* < 0.001) compared to the medical students (52%, CI 39% to 64%, I^2^ = 87.90, *p* < 0.001). These differences were not significant. The pooled prevalence of good knowledge was higher for the studies ranked as poor (59%, CI 38% to 79%, I^2^ = 97.05, *p* < 0.001), followed by good (58%, CI 50% to 65%, I^2^ = 82.39, *p* < 0.001), and fair (54%, CI 42% to 66%, I^2^ = 98.11, *p* < 0.001).

Sensitivity analysis of the overall knowledge on the key 5 questions revealed a similar trend for the continents and quality assessment. In the target population group, healthcare workers had better knowledge (62%, CI 52% to 72%, I^2^ = 95.88, *p* < 0.001) compared the general population (57%, CI 43% to 72%, I^2^ = 97.70, *p* < 0.001) and the medical students (58%, CI 53% to 63%, I^2^ = 19.70, *p* < 0.001).

A sensitivity analysis was done on specific domains considering the target population, results can be found in **Table 5**. The proportion of good knowledge was similar for both the general population and the healthcare workers, 55% (CI 48% to 62%, I^2^ = 78.42) and 54% (33% to 75%, I^2^ = 97.47), respectively, in the domain of knowledge on types of snakes and identification of snakes. Healthcare workers had higher knowledge about signs and symptoms of snakebite (73%, CI 64% to 83%, I^2^ = 92.29), first-aid (65%, CI 47% to 83%, I^2^ = 98.06), and knowledge about prevention (79%, CI 42% to 100%, I^2^ = 99.30). Healthcare workers had higher adequate knowledge on use of antivenom as the preferred treatment (81%, CI 61% to 1.00%, I^2^ = 97.91) compared to the general population (77%, CI 55% to 98%, I^2^ = 98.91). Knowledge concerning overall treatment strategy was assessed among the healthcare workers only and the proportion of adequate knowledge was 56% (CI 47% to 66%, I^2^ = 94.87).

## Discussion

Snakebite envenoming is a neglected tropical disease (NTD) that places a significant burden on many countries across South and Southeast Asia, sub-Saharan Africa, Latin America, and Australasia [11,44]. Combatting snakebite requires not only access to lifesaving antivenoms, but also communities and health systems that have the knowledge required to prevent and manage venomous snakebites. As snakebite envenoming is an ecological disease, we may speak of the production and distribution of effective antivenoms combining with knowledge of snakes and snakebite to constitute an “ecology of practice”—a composite “tool”—for mitigating the burden of this NTD [45]. This systematic review investigated community and healthcare worker knowledge and awareness on snakebite prevention and management with a view to gaining an understanding of the status of this ecology of practice in affected communities. Knowledge included signs and symptoms of snakebite, first-aid, treatment, antivenom, and preventive measures that lead to the health-seeking behaviour of the general population and the management procedure of the healthcare providers.

Data were compiled from 16 studies across 12 countries or territories, reporting 7,640 participants’ knowledge of snakebite, its treatment and management. The proportion of adequate knowledge was highest among participants from Asia (60%) compared to Africa (49%) and the Middle East (47%). All the studies included in this systematic review were from low and lower-middle-income countries in the tropical and equatorial regions of the world. No study from high-income countries met the inclusion criteria for this systematic review, though it is noted that some parts of North America [46,47] and Australasia [11,48] are prone to snakebite envenoming and fatality.

The study demonstrated a pooled adequate knowledge score of 56% among the study population. However, to account for the differences in how the included studies assessed knowledge of snakebite management and mitigation, we also undertook a pooled assessment of adequate knowledge. This enabled more direct comparison and encompassed 6 selective knowledge domains: antivenom as the preferred treatment, overall treatment, symptoms of snakebite, first-aid, snake identification/types, and prevention. When the pooled adequate knowledge for the total of domains was calculated, it was found to be higher (60%) compared to the overall pooled knowledge score of 56%.

Participants had relatively high knowledge of snakebite prevention (73%), though lower knowledge of specific snake species and how to identify them (54%). Knowledge of how to effectively prevent snakebite is fundamental to reducing the morbidity and mortality of envenoming in snakebite endemic regions. Preventative measures are most effective when they consider the local context in which snakebites occur. This includes the circumstances of how most venomous bites occur, where venomous species are likely to be encountered and what times of the day, night, or year they’re most active. For example, in south Asia, venomous kraits (*Bungarus* spp.) bite almost exclusively at night when people are sleeping on the ground in their homes [49]. In these areas, sleeping under mosquito nets considerably reduce the risk of nocturnal bites [50]. Sleeping on raised beds has also shown some promise in preventing snakebites [51]. In other snakebite endemic regions, the use of long pants and closed footwear is thought to be an effective strategy for preventing snakebites [51,52]. Again, such context-specific knowledge highlights the importance of adopting an ecological or contextual “stance”: community education focused on reducing the risk of bites via the cultivation of an ecology of practice likely offers an effective means of mitigating or reducing the impact of snakebite in these areas [53]. Such measures are also key to ensuring that local snake populations are not negatively impacted by their proximity to human communities. Snakes remain an important part of the local ecology and act to control rodent populations that are often detrimental to local agriculture and human health.

In the studies reviewed, knowledge regarding snakebite prevention was relatively high among both the general population (79%) and healthcare workers (68%). These findings were in line with previous studies conducted among doctors and communities in snakebite prevalent countries [2,40]. However, it is worth highlighting that there are no controlled studies investigating either the adequacy of the questions posed to participants in the studies—i.e., whether or not success in answering them translates into real-world knowledge of snakebite prevention—or indeed whether such knowledge in fact results in a reduction of snakebite incidents. Much research remains to be conducted in this area.

Effective first aid and treatment methods are essential to reduce the mortality and morbidity of snakebite patients. According to the WHO, appropriate first aid involves moving the person away from the area where the bite occurred, remove constricting clothing and jewellery, immobilizing the person and splinting the bitten limb to reduce movement. In some cases—e.g., within Australasia—pressure immobilisation bandages may be recommended. The person should be reassured and closely monitored as they are transported to a nearby health facility. Here, we found that participants had a reasonable knowledge of first aid overall (57%). The proportion of good knowledge about first aid was higher among healthcare workers (65%) compared to the general population (46%). Considerable gaps in knowledge still exist regarding the appropriate the first aid treatment given to snakebite victims, including the evidence base for any particular treatment. Research on the effectiveness of first aid measures is required alongside community education to ensure that at-risk individuals are aware of the appropriate first aid response for snakes’ endemic in their areas.

Approximately 33% of the participants were found to rely on “traditional” or “alternative” treatments for snakebite. These treatments often have negligible scientific support [22,54,55] and in some cases (e.g., bite site scarification, tourniquets) may be actively harmful [56]. In some instances, it may be possible to incorporate traditional healers and healing practices into the management of snakebite, particularly if they do not impede or delay the administration of antivenoms and other appropriate treatments [57]. For example, traditional healers could be provided with training regarding how to appropriately detect signs of envenoming and encouraged to use their position within communities to refer patients to local health facilities for appropriate patient management.

It is impossible to definitively state ahead of time whether a bite from any given snake or snake species will cause significant sequelae. Thus, a bite from any venomous or unidentified species of snake must be considered a life-threatening emergency. Given the diversity of potentially dangerous snake (including >700 species worldwide with high-pressure venom systems and a number of “non-front-fanged” species), it is of paramount importance for healthcare workers and those at risk to be able to recognise the signs and symptoms of envenoming from different venomous species in snakebite-endemic regions. Previous studies have indicated that inadequate knowledge of snake identification and snakebite symptoms can lead to increased mortality due to envenoming [58,59]. The results of this study indicate that overall, 54% of the participants answered questions regarding types of snakes or identification of snakes correctly. Knowledge of snake identification was found to be similar among both the general population and healthcare workers. Research has also previously indicated that healthcare workers lack knowledge and training in snake identification by signs and symptoms, and this lack of awareness results in ineffective snakebite management and increased medical errors [60,61]. Developing methods for accurate identification of species-specific envenoming and its proper management (i.e., administration of appropriate antivenom) by qualified healthcare workers proves significant in reduced mortality and morbidity associated with snakebites [62,63]. Our study results showed that there was a high level of pooled knowledge among healthcare workers (71%) in identifying the signs and symptoms after venomous snakebites. These findings are promising though the small sample size here must be noted.

Where snakebites cannot be prevented, ready access to safe and effective antivenoms and appropriate patient management from skilled medical practitioners can be lifesaving. Following a snakebite, pathological sequelae may rapidly progress towards life-threatening consequences, and appropriate medical treatment should be sought without delay. Where broad spectrum antivenom products are unavailable, effective management of snakebite patients requires specialised knowledge to administer appropriate antivenoms. Interestingly, we found that the general population had better knowledge regarding appropriate snakebite treatments (71%) than their counterparts in healthcare (59%). However, it should be noted that the public were only assessed on the appropriate use of antivenom in general. Studies have consistently reported a low level of knowledge among healthcare professionals on appropriate snakebite management. With similar gaps in knowledge evident in health sectors of developed countries, such as the United Kingdom and Hong Kong, and in low-middle income countries, such as Nigeria, Bangladesh, Lao People’s Democratic Republic, and Cameroon [2,15,16,35,64,65].

To the best of our knowledge, this is the first systematic review and meta-analysis aiming to assess knowledge about snakebite and its prevention and management among the general population and healthcare workers. This systematic review has several strengths and limitations. Firstly, all included studies were cross-sectional surveys and quantitative in nature, which means that they were unable to represent any causal associations and the exclusion of qualitative studies may limit the scope and potential of this study. Secondly, it should also be noted that the heterogeneity in the evaluated outcomes was high. While a range of sensitivity analyses were performed to identify the sources of heterogeneity, it is noted that a more standardised approach to the collection of snakebite knowledge data would enable more robust findings to be found and stronger conclusions to be drawn. A wide heterogeneity was also observed in the pooled knowledge prevalence. This was partly explained by the differences in the questionnaire contents, measurement and scoring systems. The studies ranked as good in the NHLBI quality assessment tool were found to be more homogeneous. Thirdly, this review excluded articles published in languages other than English. Given the high rates of snakebite across Southeast Asia, sub-Saharan Africa, Latin America, and the Middle East, it is possible that studies which could have provided valuable insight here were instead excluded. Additionally, due to the lack of sufficient subgroup information, this review was unable to assess the knowledge in age, gender, or education groups.

## Conclusion

The pooled proportion of good knowledge about snakebites and its management among the general population and healthcare workers was 56%. According to the survey methods utilised, participants had relatively higher knowledge regarding snakebite prevention (73%), though lower knowledge regarding snake identification (54%). These data suggest that there is a significant need for greater awareness and education of the appropriate preventative and treatment measures for snakebite for both communities and healthcare workers alike in snakebite endemic regions. Such education should include knowledge regarding local venomous snake species, their movement and behaviours; signs and symptoms of envenomation; primary first-aid for appropriate patient management and the importance of rapid health-seeking behaviours and appropriate snakebite treatment administration to reduce the burden of snakebite mortality and morbidity. The degree to which this knowledge translates into an opposite ecology of practice that contributes to the reduction of snakebite remains to be assessed. Additional studies on heterogeneous populations from other parts of the world are also needed.

**Learning Points**

- Snakebite envenoming is a neglected tropical disease (NTD) that places a significant burden on communities and health systems across the globe.
- Adequate knowledge about snakebite and snakebite management among the general population and healthcare workers was 56%.
- There is a significant need for greater awareness and education in both the general population and among healthcare workers to ensure that appropriate preventative and patient management techniques are being utilised in snakebite endemic regions.
- Future studies should look to standardise measures assessing snakebite knowledge and patient management to enable direct comparisons and ensure successful interventions are transferable to other snakebite endemic regions.
- The degree to which this knowledge translates into an opposite ecology of practice that contributes to the reduction of snakebite remains to be assessed.

**Key Papers**

1. Gutiérrez JM, Calvete JJ, Habib AG, Harrison RA, Williams DJ, Warrell DA. Snakebite envenoming. Nat Rev Dis Primers. 2017;3(1):1–21.
2. Longbottom J, Shearer FM, Devine M, Alcoba G, Chappuis F, Weiss DJ, et al. Vulnerability to snakebite envenoming: a global mapping of hotspots. Lancet. 2018;392(10148):673–84.
3. Kasturiratne A, Wickremasinghe AR, de Silva N, Gunawardena NK, Pathmeswaran A, Premaratna R, et al. The global burden of snakebite: a literature analysis and modelling based on regional estimates of envenoming and deaths. PLoS Med. 2008;5(11):e218.
4. Michael GC, Grema BA, Aliyu I, Alhaji MA, Lawal TO, Ibrahim H, et al. Knowledge of venomous snakes, snakebite first aid, treatment, and prevention among clinicians in northern Nigeria: a cross-sectional multicentre study. Trans R Soc Trop Med Hyg. 2018;112(2):47–56.
5. Pandey DP, Khanal BP. Inclusion of incorrect information on snakebite first aid in school and university teaching materials in Nepal. J Toxicol Environ Health Sci. 2013;5(3):43–51.

## Supporting information

S1 PRISMA Checklist Checklist addressing the introduction, methods, results, and discussion sections of the systematic review report. (DOCX)

S1 Table Search strategy: Ovid Medline (R). (DOCX)

S2 Table Quality assessment results for the included studies following the National Heart, Lung and Blood Institute (NHLBI) quality assessment for observational cohort and cross-sectional studies. (DOCX)
