# Supplementary material for: Assessing knowledge and awareness regarding snakebite and management of snakebite envenoming in healthcare workers and the general population: A systematic review and meta-analysis
Source: PLoS Negl Trop Dis. 2023 Feb 9;17(2):e0011048. doi: 10.1371/journal.pntd.0011048 (PMC9910687; doi:10.1371/journal.pntd.0011048)
Supplement: S1 Table — (DOCX) [file pntd.0011048.s002.docx]

S1 Table. Search strategy: Ovid Medline (R)

| **#** | **Searches** |
| --- | --- |
| 1 | exp Snake Venoms/ or (snake* adj3 venom*).tw. |
| 2 | Crotalid Venoms/ or Snake Bites/ or Elapid Venoms/ or (((crotalid or elapid) adj3 venom*) or (snake bite* or snakebite*)).tw. |
| 3 | exp Scorpion Venoms/ or (scorpion* adj3 venom*).tw. |
| 4 | exp Spider Venoms/ or ((spider* adj3 venom*) or spider bite*).tw. |
| 5 | exp Arthropod Venoms/ or ((arthropod* or ant or ants or bee or bees or wasp*) adj3 venom*).tw. |
| 6 | exp Cnidarian Venoms/ or exp Marine Toxins/ or exp Marine Toxins/ or ((cnidarian or marine or jellyfish or fish) adj3 venom*).tw. |
| 7 | 1 or 2 or 3 or 4 or 5 or 6 |
| 8 | exp knowledge/ or knowledge.tw. |
| 9 | exp attitude/ or attitude*.tw. |
| 10 | exp practice/ or practice*.tw. |
| 11 | exp Awareness/ |
| 12 | (knowledge, attitude and practice).mp. [mp=title, abstract, original title, name of substance word, subject heading word, floating sub-heading word, keyword heading word, organism supplementary concept word, protocol supplementary concept word, rare disease supplementary concept word, unique identifier, synonyms] |
| 13 | 8 or 9 or 10 or 11 or 12 |
| 14 | 7 and 13 |
| 15 | limit 14 to yr="2000 -Current" |
| 16 | limit 15 to English language |
