# Supplementary material for: Assessing knowledge and awareness regarding snakebite and management of snakebite envenoming in healthcare workers and the general population: A systematic review and meta-analysis
Source: PLoS Negl Trop Dis. 2023 Feb 9;17(2):e0011048. doi: 10.1371/journal.pntd.0011048 (PMC9910687; doi:10.1371/journal.pntd.0011048)
Supplement: S2 Table — (DOCX) [file pntd.0011048.s003.docx]

| Criteria | Ahsan et al , 2017^[1]^ | Ameade et al, 2021^[2]^ | Bala et al, 2021^[3]^ | Chincholikar et al, 2014^[4]^ | Chen et al, 2016^[5]^ | Mahmood et al, 2019^[6]^ | Pandey et al, 2016^[7]^ | Silva et al, 2014^[8]^ | Sapkota et al, 2020^[9]^ | Chaitnaya et al, 2021^[10]^ | Inthanomchanh et al, 2017^[11]^ | Kharusha et al^[12]^ | Micheal et al, 2018^[13]^ | Oomos et al, 2020^[14]^ | Subedi et al, 2018^[15]^ | Sulaiman et al, 2020^[16]^ |
| --- | --- | --- | --- | --- | --- | --- | --- | --- | --- | --- | --- | --- | --- | --- | --- | --- |
| 1. Was the research question or objective in this paper clearly stated? | Yes | Yes | Yes | Yes | Yes | Yes | Yes | Yes | Yes | Yes | Yes | Yes | Yes | Yes | Yes | Yes |
| 2. Was the study population clearly specified and defined? | Yes | Yes | Yes | Yes | Yes | Yes | Yes | Yes | Yes | Yes | Yes | Yes | Yes | Yes | Yes | Yes |
| 3. Was the participation rate of eligible persons at least 50%? | NR | Yes | Yes | NR | Yes | Yes | NR | NR | NR | NR | NR | Yes | Yes | Yes | NR | Yes |
| 4. Were all the subjects selected or recruited from the same or similar populations (including the same time period)? Were inclusion and exclusion criteria for being in the study prespecified and applied uniformly to all participants? | Yes | Yes | Yes | Yes | Yes | Yes | Yes | Yes | Yes | Yes | Yes | Yes | Yes | Yes | Yes | Yes |
| 5. Was a sample size justification, power description, or variance and effect estimates provided? | No | Yes | Yes | No | No | No | No | No | No | No | No | No | Yes | Yes | No | Yes |
| 6. For the analyses in this paper, were the exposure(s) of interest measured prior to the outcome(s) being measured? | NA | NA | NA | NA | NA | NA | NA | NA | NA | NA | NA | NA | NA | NA | NA | NA |
| 7. Was the timeframe sufficient so that one could reasonably expect to see an association between exposure and outcome if it existed? | NA | NA | NA | NA | NA | NA | NA | NA | NA | NA | NA | NA | NA | NA | NA | NA |
| 8. For exposures that can vary in amount or level, did the study examine different levels of the exposure as related to the outcome (e.g., categories of exposure, or exposure measured as continuous variable)? | Yes | Yes | Yes | Yes | Yes | Yes | Yes | Yes | Yes | Yes | Yes | Yes | Yes | Yes | Yes | Yes |
| 9. Were the exposure measures (independent variables) clearly defined, valid, reliable, and implemented consistently across all study participants? | Yes | Yes | Yes | Yes | Yes | Yes | Yes | Yes | Yes | Yes | Yes | Yes | Yes | Yes | Yes | Yes |
| 10. Was the exposure(s) assessed more than once over time? | NA | NA | NA | NA | NA | NA | NA | NA | NA | NA | NA | NA | NA | NA | NA | NA |
| 11. Were the outcome measures (dependent variables) clearly defined, valid, reliable, and implemented consistently across all study participants? | Yes | Yes | Yes | No | Yes | Yes | Yes | Yes | Yes | Yes | Yes | Yes | Yes | Yes | Yes | Yes |
| 12. Were the outcome assessors blinded to the exposure status of participants? | NA | NA | NA | NA | NA | NA | NA | NA | NA | NA | NA | NA | NA | NA | NA | NA |
| 13. Was loss to follow-up after baseline 20% or less? | NA | NA | NA | NA | NA | NA | NA | NA | NA | NA | NA | NA | NA | NA | NA | NA |
| 14. Were key potential confounding variables measured and adjusted statistically for their impact on the relationship between exposure(s) and outcome(s)? | No | No | Yes | No | No | No | No | No | No | No | No | No | No | No | No | No |
|  | Poor | Fair | Good | Poor | Poor | Fair | Good | Fair | Fair | Fair | Fair | Good | Good | fair | Fair | Fair |

S2 Table. Quality assessment results for the included studies following the National Heart, Lung and Blood Institute (NHLBI) quality assessment for observational cohort and cross-sectional studies

**References:**

1. Ahsan HN, Rahman MR, Amin R, Chowdhury EH. Knowledge of Snake bite management among health service providers at a rural Community of Bangladesh. Journal of Current and Advance Medical Research. 2017;4(1):17-22.

2. Ameade EPK, Bonney I, Boateng ET. Health professionals' overestimation of knowledge on snakebite management, a threat to the survival of snakebite victims—a cross-sectional study in Ghana. PLoS neglected tropical diseases. 2021;15(1):e0008756.

3. Bala AA, Jatau AI, Yunusa I, Mohammed M, Mohammed A-KH, Isa AM, et al. Knowledge assessment of anti-snake venom among healthcare practitioners in northern Nigeria. Therapeutic Advances in Infectious Disease. 2021;8:20499361211039379.

4. Chaaithanya IK, Abnave D, Bawaskar H, Pachalkar U, Tarukar S, Salvi N, et al. Perceptions, awareness on snakebite envenoming among the tribal community and health care providers of Dahanu block, Palghar District in Maharashtra, India. PloS one. 2021;16(8):e0255657.

5. Chen C, Gui L, Kan T, Li S, Qiu C. A survey of snakebite knowledge among field forces in China. International journal of environmental research and public health. 2017;14(1):15.

6. Chincholikar SV, Bandana P, Swati R. Awareness of Snake bite and its first aid management in rural areas of Maharashtra. Indian Journal of Community Health. 2014;26(3):311-5.

7. Inthanomchanh V, Reyer JA, Blessmen J, Phrasisombath K, Yamamoto E, Hamajima N. Assessment of knowledge about snakebite management amongst healthcare providers in the provincial and two district hospitals in Savannakhet Province, Lao PDR. Nagoya journal of medical science. 2017;79(3):299.

8. Kharusha IK, Sulaiman SS, Samara AM, Al-Jabi SW, Zyoud SeH. Assessment of knowledge about first aid methods, diagnosis, and management of snakebite among nursing students: a cross-sectional study from Palestine. Emergency Medicine International. 2020;2020.

9. Mahmood MA, Halliday D, Cumming R, Thwin KT, Myitzu M, White J, et al. Inadequate knowledge about snakebite envenoming symptoms and application of harmful first aid methods in the community in high snakebite incidence areas of Myanmar. PLoS neglected tropical diseases. 2019;13(2):e0007171.

10. Michael GC, Grema BA, Aliyu I, Alhaji MA, Lawal TO, Ibrahim H, et al. Knowledge of venomous snakes, snakebite first aid, treatment, and prevention among clinicians in northern Nigeria: a cross-sectional multicentre study. Transactions of The Royal Society of Tropical Medicine and Hygiene. 2018;112(2):47-56.

11. Ooms GI, van Oirschot J, Waldmann B, von Bernus S, van den Ham HA, Mantel-Teeuwisse AK, et al. The current state of snakebite care in Kenya, Uganda, and Zambia: healthcare workers' perspectives and knowledge, and health facilities' treatment capacity. American Journal of Tropical Medicine and Hygiene. 2021;104(2).

12. Pandey DP, Khanal BP. Inclusion of incorrect information on snakebite first aid in school and university teaching materials in Nepal. Journal of Toxicology and Environmental Health Sciences. 2013;5(3):43-51.

13. Sapkota S, Pandey DP, Dhakal GP, Gurung DB. Knowledge of health workers on snakes and snakebite management and treatment seeking behavior of snakebite victims in Bhutan. PLoS neglected tropical diseases. 2020;14(11):e0008793.

14. Silva A, Marikar F, Murugananthan A, Agampodi S. Awareness and perceptions on prevention, first aid and treatment of snakebites among Sri Lankan farmers: a knowledge practice mismatch? Journal of Occupational Medicine and Toxicology. 2014;9(1):1-3.

15. Subedi N, Paudel IS, Khadka A, Shrestha U, Mallik VB, Ankur K. Knowledge of first aid methods and attitude about snake bite among medical students: a cross sectional observational study. Journal of occupational medicine and toxicology. 2018;13(1):1-7.

16. Sulaiman SS, Kharusha IK, Samara AM, Al-Jabi SW, Sa’ed HZ. An assessment of medical students’ proficiency in the diagnosis and management of snakebites: a cross-sectional study from Palestine. Journal of occupational medicine and toxicology. 2020;15(1):1-11.
